# Cancer patients with sepsis admitted to a specialized onco-medical ICU: incidence, ICU course and outcome

**DOI:** 10.1186/cc10381

**Published:** 2011-10-27

**Authors:** D Juneja, O Singh, Y Javeri, P Nasa, P Kumar

**Affiliations:** 1 Department of Critical Care Medicine, Max Super Speciality Hospital, Saket, New Delhi, India

## Introduction

While cancer patients are known to be at higher risk for infection and subsequent complications, there is a scarcity of data regarding incidence and outcome of septic cancer patients admitted to ICUs. Hence, we aimed to assess the incidence of cancer patients admitted with sepsis to an onco-medical ICU and study their ICU course and outcome.

## Methods

Data were collected prospectively from all cancer patients admitted to a specialized onco-medical ICU of a tertiary care hospital, over a period of 6 months. Sepsis was defined as per the international guidelines. Cancer patients were divided into two groups on the basis of presence of sepsis on ICU admission and compared with regard to their need for organ support, length of ICU stay and mortality. Severity of illness was assessed by APACHE II score and organ failure by SOFA score. Qualitative data were analyzed using the chi-squared test or Fisher exact test as appropriate and quantitative data were analyzed using Student's *t *test. *P *< 0.05 was considered significant.

## Results

Out of 104 cancer patients admitted during the study period, 43 (41.3%) patients were admitted with sepsis. Even though there was no difference in age (*P *= 0.13), sex (*P *= 0.382) and presence of metastasis (*P *= 0.314) among the septic and nonseptic groups of patients, septic patients had comparatively higher admission APACHE II (21.4 ± 7 vs. 18.21 ± 6.9; *P *= 0.023) and SOFA (6.4 ± 3.8 vs. 4.56 ± 3; *P *= 0.007) scores, required invasive mechanical ventilation (65.1% vs. 19.7%; *P *= 0.000) and vasopressor support (74.4% vs. 19.7%; *P *= 0.000) more often, had a longer ICU stay (10.77 ± 8.4 vs. 7.44 ± 5.5; *P *= 0.017) and had a higher ICU mortality (62.8% vs. 18%; *P *= 0.000). The odds ratio and relative risk of death for septic cancer patients were 7.67 (95% CI = 3.121 to 18.85) and 3.482 (95% CI = 1.945 to 6.234).

## Conclusion

A significant proportion of cancer patients are admitted with evidence of sepsis. These patients were generally sicker, required more intensive organ support, and had a longer ICU stay and a higher ICU mortality than those cancer patients admitted to ICU with other acute problems.

